# Balancing Tumor Response and Rejection Risk After Pre-Transplant Immunotherapy: A Scoping Review

**DOI:** 10.3390/cancers18081284

**Published:** 2026-04-18

**Authors:** Berkay Demirors, Matthew Yu-Sheng Lin, Francis J. Spitz, Abiha Abdullah, Vrishketan Sethi, Michele Molinari

**Affiliations:** 1 Department of Surgery, University of Pittsburgh Medical Center, Pittsburgh, PA 15213, USA; spitz.francis@medstudent.pitt.edu (F.J.S.); abdullaha2@upmc.edu (A.A.); sethiv@upmc.edu (V.S.); molinarim@upmc.edu (M.M.); 2 Department of Surgery, Kaohsiung Medical University, Kaohsiung 807378, Taiwan; yushenglin.kmumed@gmail.com

**Keywords:** immune checkpoint inhibitors, liver transplantation, hepatocellular carcinoma, cholangiocarcinoma, allograft rejection

## Abstract

**Importance:** Immune checkpoint inhibitors (ICIs) have expanded downstaging options for hepatocellular carcinoma (HCC) and cholangiocarcinoma (CCA), enabling bridging to liver transplantation (LT). However, the immunologic consequences of pre-transplant checkpoint blockade, particularly the risk of allograft rejection mediated by persistent T-cell activation, remain insufficiently characterized, creating a critical knowledge gap at the intersection of immuno-oncology and transplant medicine. **Objective:** To synthesize current evidence on oncologic outcomes, rejection risk, washout intervals, donor-type considerations, and immunosuppression strategies in LT recipients with pre-transplant ICI exposure. **Evidence Review:** A PRISMA-ScR-guided review was conducted using MEDLINE, Embase, Cochrane Library, and Web of Science from January 2015 through December 2025. Studies reporting outcomes in adult LT recipients with documented pre-transplant ICI exposure for HCC or CCA were included. Methodological quality was descriptively assessed using the Newcastle-Ottawa Scale and JBI tools. Given study heterogeneity, findings were narratively synthesized. **Findings:** Thirty studies were included. In HCC, neoadjuvant ICI therapy achieved downstaging to Milan criteria in 75.6% of candidates in the largest multicenter cohort (n = 117), with complete pathologic response rates ranging from 23.8% to 40%. Rejection rates ranged from 16.3% to 20.2% in large series but increased to 56.3% with short washout intervals. Washout intervals exceeding 50 days were associated with rejection rates approaching non-ICI controls, while an individual patient meta-analysis of 91 patients estimated each additional week of washout was associated with approximately 8% reduction in rejection risk, suggesting that approximately 94 days may be required to achieve a rejection probability of 20% or less. Rejection occurred at a median of 7–10 days post-transplantation, earlier than typical acute cellular rejection. Three-year overall survival exceeded 85.3% in major cohorts. Donor type was not consistently associated with rejection after adjustment for washout duration. CCA data remain limited. Immune-related adverse events during ICI therapy were associated with increased post-transplant rejection risk. **Conclusions:** Pre-transplant ICI therapy may expand transplant eligibility in advanced hepatobiliary malignancies but carries time-dependent rejection risk. Current evidence supports a minimum washout interval of at least 50 days, with emerging data favoring 90–94 days when feasible. Prospective multicenter studies, biomarker-guided risk stratification, and standardized immunosuppression protocols are needed to refine patient selection and optimize timing.

## Simple Summary

Immune checkpoint inhibitors (ICIs) are increasingly used to downstage hepatocellular carcinoma and cholangiocarcinoma before liver transplantation. However, these therapies may increase the risk of post-transplant rejection due to persistent immune activation. This scoping review summarizes current evidence regarding oncologic outcomes, rejection risk, washout intervals, and transplant considerations in patients receiving ICIs prior to liver transplantation. Our findings highlight the importance of adequate washout intervals and multidisciplinary decision-making to balance oncologic benefit with graft safety.

## 1. Introduction

### 1.1. Background and Rationale

Hepatocellular carcinoma (HCC) and cholangiocarcinoma (CCA) represent the predominant primary hepatic malignancies, accounting for over one million new diagnoses and 900,000 deaths annually worldwide [1]. HCC ranks as the sixth most common malignancy globally and the third leading cause of cancer-related mortality, while CCA, though less prevalent, carries an even worse prognosis with 5-year survival rates below 10% for advanced disease [1]. The global burden continues to rise, driven by increasing metabolic dysfunction-associated steatotic liver disease (MASLD) in Western populations and persistent viral hepatitis in endemic regions.

Liver transplantation (LT) remains the only curative-intent treatment for selected patients, achieving 5-year survival rates of 70–80% for HCC within Milan criteria (Milan criteria: single lesion ≤ 5 cm, or up to three lesions each ≤3 cm, with no macrovascular invasion or extrahepatic disease) [2,3]. However, many patients present beyond conventional transplant thresholds and historically have been limited to systemic therapies with modest survival benefit. These malignancies have distinct histologic subtypes, including intrahepatic CCA (iCCA), perihilar CCA (pCCA), and mixed HCC-CCA.

This therapeutic landscape has been fundamentally reshaped by the advent of immune checkpoint inhibitors (ICIs). ICIs have demonstrated durable tumor control and, in selected cases, facilitated downstaging to accepted tumor-burden thresholds for LT, most commonly the Milan or UNOS downstaging criteria [4,5,6]. Their integration into pre-transplant management represents a major shift in hepatobiliary oncology. Yet this advance introduces a fundamental paradox: checkpoint pathways required for tumor immune escape are also essential for maintaining allograft tolerance after transplantation [7]. This mechanistic conflict has created a clinical dilemma that requires evidence-based guidance on patient selection, timing, and post-transplant management.

### 1.2. The Evolving Landscape of ICI Therapy in Liver Malignancy

The approval of ICIs has redefined systemic therapy for HCC and CCA. In IMbrave150, atezolizumab plus bevacizumab improved survival compared with sorafenib (median OS 19.2 vs. 13.4 months; HR 0.66) [8,9], while the HIMALAYA trial confirmed the efficacy of tremelimumab plus durvalumab in advanced HCC, with sustained survival benefit [10,11].

In biliary tract cancers, the TOPAZ-1 trial established durvalumab plus gemcitabine-cisplatin as a new standard of care (median OS 12.9 vs. 11.3 months; HR 0.76) [12,13]. These advances have expanded the role of ICIs into neoadjuvant and bridging settings, where tumor response may facilitate downstaging to transplant eligibility in selected patients [4,5,6].

### 1.3. The Transplant Dilemma: Oncologic Benefit vs. Immunologic Risk

The efficacy of ICIs in hepatobiliary malignancies reflects the immunosuppressive tumor microenvironment common to both HCC and CCA, including high levels of PD-L1 expression, exhausted T cells, and regulatory immune populations. Checkpoint blockade restores antitumor immunity; however, reactivated effector T cells may persist and subsequently recognize donor alloantigens following transplantation [7,14].

The PD-1/PD-L1 pathway exerts opposing functions in cancer immunotherapy and transplant tolerance. Within tumors, PD-L1 engagement induces T-cell exhaustion and immune escape. Blockade of this interaction reinvigorates cytotoxic T-cell function and promotes tumor clearance. However, the same pathway is critically involved in maintaining peripheral tolerance to alloantigens. PD-L1 expression on donor tissue downregulates alloreactive T-cell responses, supports regulatory T-cell activity, and prevents allograft rejection. Experimental models demonstrate that PD-L1-deficient grafts undergo rapid rejection and that checkpoint blockade accelerates graft loss in murine systems.

Checkpoint-mediated immune activation is not immediately reversible upon therapy cessation. The pharmacokinetic half-lives of commonly used ICIs range from 15 to 27 days, but pharmacodynamic effects persist substantially longer [15,16]. PD-1 receptor occupancy on circulating T cells exceeds 70% for up to 2 months after infusion and may remain above 50% for more than 200 days following repeated dosing [16]. This sustained receptor engagement and memory T-cell formation creates a prolonged period of immunologic vulnerability extending beyond measurable serum drug levels.

Clinically, this mechanistic tension has been documented in reports of severe and occasionally fatal allograft rejection in ICI-exposed transplant recipients [14,17,18]. Early cases of fatal hepatic necrosis following nivolumab bridging therapy highlighted the potential risks, while subsequent reports underscored uncertainty regarding safe transplantation timing and the need for evidence-based guidance on washout duration and immunosuppression strategies [18,19].

This scoping review synthesizes current evidence on pre-transplant ICI exposure in LT candidates with HCC and CCA, focusing on oncologic efficacy, rejection risk, optimal washout intervals, donor considerations, and immunosuppression approaches.

## 2. Methods

### 2.1. Protocol and Registration

This scoping review was conducted in accordance with the Preferred Reporting Items for Systematic Reviews and Meta-Analyses extension for Scoping Reviews (PRISMA-ScR) guidelines and the Joanna Briggs Institute (JBI) methodology for scoping reviews. A scoping review design was selected because the objective is to map existing evidence across heterogeneous study designs, rather than to perform only quantitative synthesis.

### 2.2. Eligibility Criteria

Studies were eligible if they included adult patients (≥18 years) with HCC, intrahepatic CCA (iCCA), perihilar CCA (pCCA), or mixed HCC-CCA who underwent LT following ICI exposure. ICI exposure was defined as receipt of at least one dose of any PD-1 inhibitors (nivolumab, pembrolizumab, camrelizumab, sintilimab, tislelizumab, toripalimab), PD-L1 inhibitors (atezolizumab, durvalumab, avelumab), or CTLA-4 inhibitors (ipilimumab, tremelimumab), administered as monotherapy or combination therapy prior to LT.

Studies were required to report at least one of the following outcomes: tumor response, (as defined by mRECIST or RECIST v1.1), downstaging success (defined as meeting Milan criteria [single lesion ≤5 cm or up to 3 lesions each ≤3 cm] or UNOS downstaging criteria as specified in each study), rejection incidence or severity, graft survival, disease-free survival (DFS), OS, or infectious complications.

Eligible study designs included randomized controlled trials, prospective and retrospective cohort studies, case–control studies, and case series with three or more patients. Case reports were included for qualitative context only and are clearly identified as such.

Peer-reviewed publications in English or with available English translation, published from January 2015 through December 2025, were included. Conference abstracts, preclinical studies, reviews, editorials, and commentaries were excluded from the primary evidence set but were used for contextual reference and are labeled as “context-only sources” where cited.

### 2.3. Search Strategy and Study Selection

MEDLINE, Embase, Cochrane Library, Web of Science, and ClinicalTrials.gov were searched from inception through December 2025 using terms related to ICIs, LT, and HCC or CCA. Reference lists of included studies were manually screened for additional publications. Three independent reviewers screened titles, abstracts, and full texts using Covidence software (https://www.covidence.org/, Veritas Health Innovation, Melbourne, Australia; web-based platform), with disagreements resolved by consensus.

### 2.4. Data Extraction and Analysis

Data extracted included study characteristics, patient demographics, ICI regimens, washout intervals, transplant variables, rejection outcomes, and survival endpoints.

Methodological quality was descriptively assessed using the Newcastle-Ottawa Scale (NOS) for cohort studies and the JBI Critical Appraisal Checklist for case series. For cohort studies, NOS scores were assigned across three domains: selection (maximum 4 points), comparability (maximum 2 points), and outcome (maximum 3 points); scores ≥ 7/9 were considered indicative of good methodological quality. For case series, the JBI Critical Appraisal Checklist (10-item version) was applied, with item-level responses mapped descriptively. Consistent with scoping review methodology, these assessments informed descriptive contextualization of the evidence base and were not used to exclude studies.

Given the heterogeneity of included studies, sample size, outcome definitions, and immunosuppression protocols, findings were synthesized narratively. Quantitative data are presented as ranges across studies, with specific thresholds attributed to their source. All quantitative estimates derived from observational data should be considered as hypothesis-generating due to the predominance of retrospective designs and potential selection bias.

## 3. Results

### 3.1. Study Selection and Characteristics

**Figure 1:**
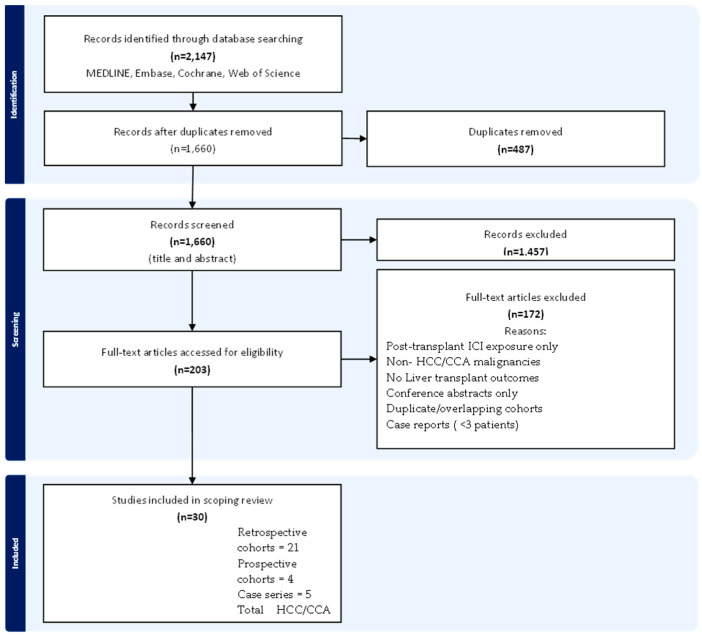
PRISMA flow diagram of study selection process. Systematic literature search and study selection process following PRISMA-ScR guidelines. The search across MEDLINE, Embase, Cochrane Library, and Web of Science identified 2147 records. After duplicate removal and screening, 30 studies encompassing >1200 HCC/CCA patients who received ICI therapy prior to liver transplantation were included in the final scoping review. Abbreviations: ICI, immune checkpoint inhibitor; HCC, hepatocellular carcinoma; CCA, cholangiocarcinoma; PRISMA-ScR, Preferred Reporting Items for Systematic Reviews and Meta-Analyses extension for Scoping Reviews.

The search identified 2147 records; 30 studies met the inclusion criteria, encompassing over 1200 ICI-exposed LT recipients (Figure 1). One conference abstract was retained as context-only [20]. Studies were published between 2020 and 2025, originating from North America (n = 10), Europe (n = 8), Asia (n = 10), and multicontinental collaborations (n = 2). Most were retrospective cohorts (n = 21), with four prospective cohorts and five case series. Sample sizes ranged from 4 to 386 patients [4,5,21].

HCC accounted for over 90% of cases, with limited CCA representation (43 patients across transplant-specific series). ICI regimens included PD-1 monotherapy, PD-L1 plus anti-VEGF combinations, and dual checkpoint blockade. Median washout intervals ranged from 30 to 180 days. Eleven cohort studies achieved NOS scores ≥ 7/9, though the predominance of retrospective designs limits causal inference. Key studies are summarized in Table 1.

### 3.2. ICI-Based Bridging and Downstaging Efficacy

#### 3.2.1. Hepatocellular Carcinoma

ICI-based regimens, often combined with locoregional therapies, achieved successful downstaging in candidates initially exceeding Milan criteria. In the VITALITY study (n = 117 intention-to-treat (ITT); 43 transplanted), 75.6% were successfully downstaged, with 3-year ITT survival of 71.1% and rejection incidence of 16.3% among transplanted patients [4]. Khalaf et al. reported 82% bridging success and 85.3% 3-year post-transplant survival in 87 patients [6].

Radiologic response consistently underestimated pathologic efficacy. Explant analyses revealed tumor necrosis rates of 35–88%, with complete pathologic response (0% viable tumor) in 23.8–40%, depending on the cohort and ICI regimen [4,5,23]. Ma et al. reported a 3-year OS of 88.2% among 386 patients in a global retrospective cohort [21].

Ongoing prospective trials, including the PLENTY pilot study (Lv et al., NCT04425226) and ImmunoXXL study (NCT05879328), will provide prospective validation of these combination approaches [22].

#### 3.2.2. Cholangiocarcinoma

Evidence for ICI-based bridging in CCA remains limited and preliminary. Transplant-specific data are confined to small series. Under the SYS-TARE protocol, 4 of 13 evaluated patients with unresectable liver-limited iCCA underwent transplantation, all remaining disease-free at follow-up to 73 months [29].

Neoadjuvant gemcitabine-cisplatin-durvalumab demonstrated 46.2% conversion to resection and 58.3% pathologic response in borderline resectable CCA, though these data pertain to resection rather than transplantation [30].

OPTN/HRSA guidance (2025) provides MELD exception pathways for selected iCCA cases but does not constitute formal policy. Overall, CCA transplant data remain hypothesis-generating.

### 3.3. Washout Interval and Rejection Risk

#### 3.3.1. Reported Washout Thresholds

Washout duration between the last ICI dose and transplantation emerged as the most consistent modifiable predictor of rejection. In an international cohort (n = 119), washout shorter than 30 days carried the highest risk (OR 21.3), risk remained elevated for intervals of 30–50 days (OR 9.48), and rejection rates approached non-ICI controls when washout exceeded 50 days [5].

An individual patient meta-analysis (n = 91) estimated that each additional week of washout reduced the risk of rejection by 8%, with approximately 94 days required to achieve a rejection probability of 20% or less [15]. In VITALITY, six of seven rejection episodes occurred with a washout shorter than 90 days [4].

Collectively, these data support a minimum washout of 50 days, with 90–94 days representing a more conservative target when feasible. However, all estimates derive from observational data and require individualized application.

#### 3.3.2. Pharmacokinetic Considerations

The elimination half-life of each ICI agent directly informs the minimum washout duration, as pharmacologically active concentrations persist for multiple half-lives after the last dose. PD-1 and PD-L1 inhibitors have half-lives of 18–27 days (nivolumab, pembrolizumab, atezolizumab, durvalumab); camrelizumab has a notably shorter half-life of 5.5 days, which may partially explain differential rejection rates in predominantly Asian LDLT cohorts [16].

Half-life-based modeling may better predict rejection risk than absolute day counts. Washout exceeding 1.5 drug-specific half-lives was associated with 76% lower rejection odds (OR 0.24) [17].

However, receptor occupancy persists beyond serum clearance, providing a mechanistic rationale for extended washout intervals approaching 90 days.

#### 3.3.3. Balancing Rejection Risk Against Oncologic Urgency

The clinical challenge lies in balancing allograft safety against oncologic risk during prolonged waiting. In patients with aggressive tumor biology, elevated AFP, or progression risk, reduced rejection risk from extended washout must be weighed against waitlist dropout [4]. The 50–90-day washout window represents an approximate balance for most patients, but individualization remains essential.

These factors, washout duration, tumor biology, and donor type, should be interpreted together in clinical decision-making. Shorter washout intervals (<50 days), aggressive tumor features, and limited scheduling flexibility (e.g., deceased donor transplantation) may increase rejection risk, whereas LDLT enables more precise timing to optimize washout, despite potential immunologic considerations. Accordingly, transplant timing should be individualized based on both oncologic urgency and the feasibility of achieving adequate washout.

### 3.4. Rejection in ICI-Exposed Recipients

#### 3.4.1. Incidence and Timing of Rejection Episodes

Pre-transplant ICI exposure is associated with an elevated risk of allograft rejection. Large multicenter cohorts report rejection rates of 16–20%: Moeckli et al. documented 20.2% among 119 recipients, Tabrizian et al. reported 16.3% among 43 transplanted patients in VITALITY, and Ma et al. found 17.5% among 386 patients in the global cohort study [4,5,21]. Across aggregated data, 78% of rejection episodes achieved complete recovery with standard treatment, while graft loss occurred in approximately 4% and rejection-related mortality in approximately 2% [28].

Rejection incidence varies substantially by washout duration, reaching up to 56% with short intervals [27]. In contrast, a context-only abstract reported 0% rejection with a median 4-month washout, though peer-reviewed confirmation is pending [20]. The ICI class may influence risk, but findings are inconsistent across cohorts [17,28].

Rejection in ICI-exposed recipients demonstrates distinctive features: earlier onset (median 7–10 days post-LT vs. 14–21 days in conventional ACR), predominance of CD8+ effector mobilization (lower CD4+/CD8+ ratio), and possible association with prior immune-related adverse events (irAEs) [5,21,24,25,27,28]. No validated criteria currently distinguish ICI-associated rejection histologically from conventional acute cellular rejection.

Immunosuppression strategies remain nonstandardized. Basiliximab-based induction represents the most commonly reported approach in Western DDLT cohorts [31]. Antithymocyte globulin (ATG) has been used selectively in high-risk patients, those with short washout, prior grade ≥ 2 irAEs, or LDLT, given its capacity to deplete circulating T cells prior to engraftment [31]. Higher early tacrolimus (12–15 ng/mL) has been reported at some centers during the initial post-transplant weeks [31]. mTOR inhibitor-based regimens have been associated with higher rejection rates in small series and are generally avoided early [32]. No prospective randomized evidence supports any single protocol, and institutional practice varies substantially.

#### 3.4.2. Graft Loss and Mortality

Despite higher rejection incidence, irreversible graft loss directly attributable to rejection remains relatively uncommon in the neoadjuvant ICI setting. Graft failure rates are approximately 4%, with rejection-related mortality around 2% in aggregated analyses [28]. In VITALITY, only one graft failure occurred among 43 patients [4].

However, short-term mortality is significantly elevated. Mortality at 90 days reached 14.3% in ICI-exposed recipients compared with 2.3% in controls, with rejection independently predicting early death [25]. Timing of ICI exposure critically influences outcomes: neoadjuvant use was associated with lower rejection-related mortality (18.5%) compared with post-transplant administration for recurrence (47.1%) [21].

#### 3.4.3. Tumor Recurrence and Disease-Free Survival

Tumor recurrence remains a major determinant of long-term outcomes. Aggregated data estimate HCC recurrence at approximately 24%, predominantly within two years post-LT [28]. Extrahepatic recurrence (lung, bone) appears more common than intrahepatic recurrence, though mechanistic interpretations remain speculative [28].

Complete pathologic response (23.8–40%) was associated with favorable DFS, approximating outcomes of patients initially within Milan criteria [5,6]. Residual viable tumor, microvascular invasion, and poor differentiation predict recurrence risk.

Emerging data support the oncologic benefit of combination approaches. The PLENTY pilot study reported improved recurrence-free survival with pembrolizumab plus lenvatinib (70% vs. 30%) [22]. In CCA, SYS-TARE achieved durable DFS in transplanted patients, though numbers remain small [29].

#### 3.4.4. Histopathology and Biomarkers of Rejection

ICI-associated rejection is predominantly T-cell-mediated, characterized by portal inflammation, bile duct injury, and endothelial involvement [14,28]. Most cases demonstrate moderate RAI scores; antibody-mediated rejection appears rare but incompletely assessed.

Distinct immunologic signatures have been described in ICI-associated rejection. Peripheral immune profiling reveals lower CD4+/CD8+ ratios in ICI-associated rejection, reflecting CD8+ predominance [25,27]. Exploratory biomarkers include prior irAEs and graft PD-L1 expression, though findings require replication [21,24]. Donor-derived cell-free DNA may enable early detection of subclinical graft injury before clinical manifestations, but prospective validation is needed before clinical implementation.

### 3.5. Donor Type and Graft-Related Factors

#### 3.5.1. Living Donor Liver Transplantation

LDLT offers elective scheduling that enables precise washout optimization. However, some LDLT cohorts have reported higher rejection rates (22.9–56.3%), including 50% in one series, despite prolonged washout [23]. This may reflect enhanced antigen presentation during rapid graft regeneration in the early post-transplant period when ICI-primed effector populations remain active. Importantly, rejection episodes in these cohorts resolved completely with corticosteroid therapy without graft loss. Overall survival remained acceptable, with 1–2-year OS of approximately 75% reported in one series [23].

#### 3.5.2. Donation After Brain Death and Circulatory Death

Donation after brain death (DBD) represents the traditional standard for deceased donor LT. Brain death induces systemic inflammation and endothelial activation, theoretically enhancing alloantigen presentation. Donation after circulatory death (DCD) grafts, now accounting for over 25% of deceased donor LT at many centers, are characterized by warm ischemia and greater ischemia-reperfusion injury that could theoretically amplify ICI-mediated alloimmune activation.

However, available evidence does not suggest clinically meaningful differences in rejection risk between DBD and DCD grafts when washout intervals are appropriately controlled. Guo et al. reported that DCD was not an independent risk factor for rejection (OR: 1.309, not significant), and Moeckli et al. similarly found no significant difference across donor categories after adjustment [5,26].

Thus, ICI-exposure alone should not preclude the use of DCD grafts. In deceased donor transplantation, the principal limitation is timing unpredictability rather than graft type–specific immunologic risk.

#### 3.5.3. Comparative Analysis: LDLT vs. DDLT Outcomes in ICI-Exposed Recipients

Direct comparisons remain limited by observational design, center-level practices, and differential washout optimization (Table 2). Broadly, LDLT cohorts (predominantly Asian) report higher rejection rates than DDLT cohorts (predominantly Western), despite longer median washout intervals [4,5,6,23].

This apparent paradox may reflect factors unique to LDLT: rapid graft regeneration with increased MHC class I and class II expression, greater use of camrelizumab-based regimens, whose shorter half-life (5.5 days) differs from the nivolumab and pembrolizumab predominantly used in Western DDLT cohorts, and center-level differences in immunosuppression induction protocols. These variables introduce substantial pharmacokinetic and practice-pattern confounding.

Importantly, graft loss and mortality outcomes were comparable across donor types [4,21,23]. Multivariable analyses have not consistently identified donor type as an independent predictor of rejection once washout interval and covariates are controlled [5,26].

The strategic advantage of LDLT is elective scheduling, which enables precise washout optimization. In deceased donor transplantation, structured acceptance algorithms incorporating ICI exposure and minimum washout thresholds may partially mitigate the timing unpredictability. Prospective studies with standardized washout and immunosuppression protocols are needed to clarify whether donor type independently influences rejection risk.

## 4. Current Guidelines and Consensus Statements

Several professional societies have addressed ICI use in LT candidates; however, recommendations remain heterogeneous and are constrained by limited prospective evidence (Table 3).

### Areas of Consensus and Controversy

There is general agreement that neoadjuvant ICI therapy can be considered for downstaging in carefully selected patients, with an appropriate washout period. Most societies also emphasize the need for enhanced monitoring and multidisciplinary evaluation.

However, several key controversies remain.

First, the optimal washout duration is not standardized. Guideline recommendations range from 4 to 12 weeks, whereas contemporary observational data suggest that longer intervals (50–94 days) may further mitigate rejection risk [31,33,34].

Second, whether rejection risk differs by ICI class remains unresolved. Xu et al. reported higher rejection with PD-1 monotherapy versus PD-L1 inhibitors, whereas Liu et al. found no significant class-based difference in a systematic review [17,28]. Data on CTLA-4 monotherapy in the pre-transplant setting are particularly limited, precluding class-specific recommendations [28].

Third, there is no consensus on immunosuppression strategies for ICI-exposed recipients. Although some centers report using higher early tacrolimus targets and avoiding early mTOR initiation, these approaches are based on retrospective experience and lack prospective validation or formal guideline endorsement [31,32].

Finally, no validated biomarkers for rejection risk stratification have been incorporated into guidelines. Promising exploratory candidates include prior immune-related adverse events, graft PD-L1 expression, and donor-derived cell-free DNA [21,24]. The role of post-transplant adjuvant ICI in patients with residual viable tumor likewise remains undefined.

## 5. Discussion

### 5.1. Summary of Evidence

This scoping review of 30 studies indicates that pre-transplant ICI therapy can enable downstaging in selected HCC patients beyond Milan criteria, with favorable survival in contemporary cohorts. However, these benefits are accompanied by a measurable risk of early allograft rejection [4,21].

#### Interpretation of Evidence Certainty

Given the predominance of retrospective data, findings should be interpreted cautiously. Evidence for downstaging efficacy, rejection incidence, and washout ≥ 50 days is relatively consistent across cohorts. In contrast, longer washout intervals (90–94 days), donor-type effects, immunosuppression strategies, and biomarker-based risk prediction remain supported by limited and heterogeneous data. Evidence for CCA is particularly sparse [4,5,21].

### 5.2. Clinical Implications

Several practical principles emerge.

Pre-transplant ICI exposure should not preclude transplantation in appropriately selected patients. Outcomes appear favorable when adequate washout is achieved.

Patient selection should integrate tumor biology, treatment response, washout feasibility, and immunologic risk factors, including prior irAEs. Short washout intervals or high-risk features may warrant intensified monitoring.

Clinicians should counsel patients regarding an approximate 20% rejection risk, early post-transplant onset, and generally favorable survival outcomes. Multidisciplinary evaluation remains essential [4,5,21,28].

### 5.3. Proposed Treatment Framework

#### We Propose a Four-Phase Framework

Neoadjuvant phase: ICI-based therapy ± locoregional treatment with response assessment [8,9,24].

Washout phase: Target ≥ 50 days; consider 90–94 days when feasible [5,15].

Peri-transplant phase: Risk-adapted immunosuppression (e.g., basiliximab vs. ATG, higher early tacrolimus), with close monitoring [31,32].

Long-term phase: Transition to standard immunosuppression; consider delayed mTOR introduction [32].

These strategies remain nonstandardized and require prospective validation.

### 5.4. Knowledge Gaps and Research Priorities

Key uncertainties include the impact of ICI class, optimal washout duration, and standardized immunosuppression protocols. Reliable risk stratification tools are lacking, and proposed biomarkers (e.g., irAEs, PD-L1 expression, donor-derived cfDNA) require validation. Evidence for CCA and post-transplant ICI use remains limited [17,28].

### 5.5. Design Biases Unique to This Literature

Interpretation is limited by confounding by indication, as ICI-treated patients often have more advanced disease. Transplant-based analyses may overestimate benefit by excluding patients who progress before transplantation. Heterogeneity in treatment protocols and evolving clinical practice further limits comparability.

### 5.6. Limitations

This review is limited by predominantly retrospective data, small sample sizes, and heterogeneity in treatment approaches and outcome definitions. Follow-up duration is limited, restricting assessment of long-term rejection and oncologic outcomes. Accordingly, all findings and clinical recommendations should be interpreted as hypothesis-generating rather than definitive.

## 6. Conclusions

ICIs have expanded curative-intent LT to patients with HCC and CCA previously considered ineligible. In selected patients, ICI-based therapy enables downstaging or bridging to transplantation, with survival outcomes approaching those of standard candidates.

Pre-transplant ICI exposure is associated with an increased risk of early rejection, but this risk appears modifiable with appropriate washout strategies.

These findings support the integration of ICIs into pre-transplant care with careful timing, individualized risk assessment, and multidisciplinary management. Prospective studies are needed to define optimal washout intervals, clarify class-specific risks, and establish standardized peri-transplant strategies.

## Figures and Tables

**Table 1:** Selected key studies: characteristics, key outcomes, and Newcastle-Ottawa quality scores.

Study (Author, Year)	Study Design	N	Region	Primary Indication	Washout Interval (Days)	Rejection Rate (%)	Downstaging/Response	3-yr OS (%)	Quality Score *
Prospective Cohorts									
Tabrizian et al., 2025 (VITALITY) [4]	Prospective multicenter	43/117	North America	HCC	50–90	16.3	75.6% downstaging	71.1	8/9
Lv et al., 2024 (PLENTY pilot) [22]	Prospective cohort	–	North America	HCC	–	–	–	70 (recurrence-free)	7/9
Retrospective Cohorts									
Moeckli et al., 2025 [5]	Retrospective multicenter	119	International (15 centers)	HCC	30–180	20.2	–	–	8/9
Ma et al., 2025 [21]	Retrospective global cohort	386	Global	HCC (primary)	Varied	17.5	–	88.2	8/9
Cheng et al., 2025 [23]	Retrospective cohort	10 (LDLT)	Asia (Taiwan)	HCC	149 (median)	50.0	40% cPR; 35–88% necrosis on explant	75 (1–2 yr)	6/9
Khalaf et al., 2025 [6]	Retrospective cohort	87	North America	HCC	50–94	14.9	82% bridging success	85.3	8/9
Xu et al., 2024 [17]	Retrospective multicenter	156	Asia	HCC	30–120	22.4 (PD-1: 28%; PD-L1: 18%)	–	–	7/9
Fang et al., 2024 [24]	Retrospective cohort	68	Asia	HCC	Varied	19.1	–	–	7/9
Pang et al., 2025 [25]	Retrospective matched analysis	94/94	Asia	HCC	45–90	18.0 vs. 5.4 (control)	–	–	8/9
Guo et al., 2024 [26]	Retrospective multicenter	142	Asia	HCC	50–120	23.6	–	–	7/9
Wang et al., 2023 [27]	Retrospective cohort	–	Asia	HCC	–	–	–	–	6/9
Meta-analyses & Systematic Reviews									
Liu et al., 2025 [28]	Systematic review & meta-analysis	1200+	Multicenter global	HCC/CCA	30–180	16.0–20.0 overall; 78% achieved complete response to treatment	23.8–40% cPR; 24% recurrence	85–88	–
Rezaee-Zavareh et al., 2025 [15]	Individual patient data meta-analysis	91	Multicenter	HCC	30–180	Washout-dependent (94 days = 20% rejection risk)	–	–	–
CCA-Specific Studies									
Maspero et al., 2024 (SYS-TARE) [29]	Retrospective cohort	13	International	iCCA	Varied	–	31% bridging success	100 (DFS at 73 mo)	7/9
Dong et al., 2025 [30]	Phase II study	–	Asia	CCA (borderline resectable)	–	–	46.2% conversion to surgery; 58.3% pCR	–	7/9
Context-Only Sources (not included in formal evidence synthesis)									
Esmail et al., 2025 [20]	Conference	25	North	HCC	120	0	–	–	6/9
Nordness et al., 2020 [18]	Case report	1	North America	HCC	Short	Fatal	–	–	–
Pettas et al., 2024 [19]	Case series/review	Multiple	International	HCC	Varied	Varied	–	–	5/9
Ongoing Trials (Referenced for Context)									
ImmunoXXL (NCT05879328)	Prospective RCT	Ongoing	–	HCC	–	–	–	–	–

Selected key studies reporting pre-transplant ICI exposure in liver transplant recipients. Washout intervals are presented as median or mean as reported in original publications. * Newcastle-Ottawa Scale (NOS) for cohort studies (max 9). Scores ≥ 7 indicate good quality.

**Table 2:** Comparative analysis of LDLT vs. DDLT outcomes in ICI-exposed liver transplant recipients. Direct comparison of available data stratified by donor type, with identification of key confounders limiting causal inference. Data extracted from individual study reports; no formal pooled analysis was performed.

Study	Donor Type	N	Median Washout (Days)	Rejection Rate (%)	Graft Loss/Mortality	OS
LDLT Cohorts						
Cheng et al., 2025 [23]	LDLT	10	149	50.0	0% graft loss; all rejection episodes resolved with corticosteroids	75% (1–2 yr)
Xu et al., 2024 [17] (LDLT subgroup)	LDLT	Subgroup of 156	30–120	22.9–28.0 (PD-1 subgroup)	Not separately reported for LDLT	Not separately reported
Wang et al., 2023 [27]	LDLT predominant	—	Short	56.3 (short washout)	No increased graft loss attributed to ICI exposure	—
DDLT Cohorts (predominantly or exclusively DDLT)						
Tabrizian et al., 2025 (VITALITY) [4]	DDLT (predominant)	43 transplanted/117 ITT	50–90	16.3	1/43 graft failure from rejection (2.3%)	71.1% (3-yr ITT)
Moeckli et al., 2025 [5]	DDLT (predominant)	119	30–180	20.2	Graft loss rate not separately reported; washout >50 days approached baseline rejection	—
Ma et al., 2025 [21]	Mixed (global)	386	Varied	17.5	Rejection-related mortality 18.5% (neoadjuvant) vs. 47.1% (post-LT ICI)	88.2% (3-yr)
Khalaf et al., 2025 [6]	DDLT (predominant)	87	50–94	14.9	Not separately reported	85.3% (3-yr)
Guo et al., 2024 [26]	Mixed (DBD + DCD)	142	50–120	23.6	DCD not independent risk factor vs. DBD (OR 1.309, NS)	—
Multivariable Analyses Addressing Donor Type						
Guo et al., 2024 [26]	DBD vs. DCD	142	50–120	OR 1.309 (DCD vs. DBD), not significant	Donor type not independently predictive of rejection after adjustment for washout and covariates	—
Moeckli et al., 2025 [5]	LDLT vs. DDLT	119	30–180	No significant difference across donor categories	Donor type not significant when washout adequately controlled	—
Key Confounders Limiting Direct LDLT vs. DDLT Comparison						
Graft regeneration biology	LDLT grafts undergo rapid regeneration with upregulation of MHC class I/II expression, potentially amplifying alloantigen presentation to ICI-primed T cells. This biological difference is unique to partial grafts and cannot be adjusted for in observational comparisons.					
ICI regimen confounding	LDLT cohorts (predominantly Asian) more commonly used camrelizumab (t½ 5.5 days), while DDLT cohorts (predominantly Western) used nivolumab/pembrolizumab (t½ 22–25 days). Drug-specific pharmacokinetics confound donor type comparisons.					
Scheduling advantage	LDLT enables elective timing to optimize washout, while DDLT timing depends on organ availability. This systematically different washout optimization capability confounds rejection rate comparisons between donor types.					
Center-level heterogeneity	Immunosuppression induction protocols (with/without ATG), tacrolimus target troughs, biopsy thresholds, and follow-up intensity differ substantially across LDLT-predominant (Asian) and DDLT-predominant (Western) centers.					

LDLT cohorts reported higher rejection rates (22.9–56.3%) compared with DDLT-predominant cohorts (14.9–20.2%), despite longer median washout intervals in LDLT. However, graft loss and mortality outcomes were comparable, with all LDLT rejection episodes in the Cheng et al. cohort resolving with corticosteroid therapy. Multivariable analyses by Guo et al. and Moeckli et al. did not identify donor type as an independent predictor of rejection after adjustment for washout and covariates. These findings must be interpreted cautiously, given the substantial confounders listed above. Prospective studies with standardized washout protocols and immunosuppression regimens across LDLT and DDLT are needed to determine whether donor type carries an independent immunologic risk. Strategic implication: The primary advantage of LDLT lies in converting an unpredictable surgical timeline into an elective procedure, enabling precise washout optimization. For DDLT, provisional acceptance algorithms incorporating ICI exposure status and minimum washout requirements may partially address timing unpredictability. Abbreviations: LDLT, living donor liver transplantation; DDLT, deceased donor liver transplantation; DBD, donation after brain death; DCD, donation after circulatory death; ITT, intention-to-treat; OR, odds ratio; NS, not significant; OS, overall survival; ATG, antithymocyte globulin; MHC, major histocompatibility complex.

**Table 3:** Comparison of professional society guidelines for pre-transplant ICI use.

Society/Organization	Recommended Washout Period	Key Recommendations	Monitoring/Surveillance	Immunosuppression Guidance
AASLD [33]	Minimum washout recommended (no specific duration mandated)	Neoadjuvant ICI therapy acceptable for HCC exceeding transplant criteria; multidisciplinary evaluation required	Enhanced post-transplant surveillance recommended	Not specified
EASL [14,34]	At least 4–6 weeks; preferably longer	ICIs valuable as bridging and downstaging option	Protocol biopsies suggested for enhanced rejection surveillance	Not specified
ILTS [31]	6–12 weeks minimum; longer intervals preferred when oncologically feasible	Risk stratification should consider ICI class, cycle number, and tumor response; intensified induction may be considered for high-risk cases	Enhanced monitoring with low threshold for protocol biopsy; early transplant referral when appropriate	Intensified induction (antithymocyte globulin) for high-risk patients (short washout, LDLT, irAE history)
ASCO [8,9,10,11,12,13,35]	Not specifically addressed	ICI-based regimens recommended as first-line therapy for advanced HCC and CCA; early transplant referral when appropriate	Multidisciplinary involvement emphasized	Not specified
ESMO [8,9,10,11,12,13,35]	Not specifically addressed	ICI-based regimens recommended as first-line therapy; emphasis on multidisciplinary involvement	Multidisciplinary evaluation encouraged	Not specified
Moeckli et al. & Rezaee-Zavareh et al. [5,15]	50–94 days optimal; <30 days carries highest rejection risk; 50+ days normalizes rejection to baseline	Each additional week of washout reduces rejection risk by ~8%; PD-1 half-life specific dosing (e.g., 1.5 half-lives for nivolumab ≈ 38 days)	Enhanced surveillance with protocol biopsies; immunologic biomarkers recommended	Higher tacrolimus troughs (12–15 ng/mL) initially; avoid early mTOR introduction

Summary of major transplant and oncology society recommendations regarding washout periods, monitoring strategies, and immunosuppression management for ICI-exposed liver transplant candidates. Abbreviations: AASLD, American Association for the Study of Liver Diseases; EASL, European Association for the Study of the Liver; ILTS, International Liver Transplantation Society; ASCO, American Society of Clinical Oncology; ESMO, European Society for Medical Oncology; LDLT, living donor liver transplantation; irAE, immune-related adverse event; mTOR, mechanistic target of rapamycin.

## Data Availability

The data used in this study were derived from previously published studies identified through the systematic literature search described in the Methods section. All included studies are cited in the reference list. No new patient-level datasets were generated or analyzed. Additional extracted data supporting the findings of this review are available from the corresponding author upon reasonable request.

## Abbreviations

AFP	Alpha-fetoprotein
AASLD	American Association for the Study of Liver Diseases
ASCO	American Society of Clinical Oncology
CCA	Cholangiocarcinoma
CI	Confidence interval
CTLA-4	Cytotoxic T-lymphocyte-associated protein 4
DBD	Donation after brain death
DCD	Donation after circulatory death
DFS	Disease-free survival
EASL	European Association for the Study of the Liver
ESMO	European Society for Medical Oncology
HCC	Hepatocellular carcinoma
HR	Hazard ratio
ICI	Immune checkpoint inhibitor
ILTS	International Liver Transplantation Society
iCCA	Intrahepatic cholangiocarcinoma
irAE	Immune-related adverse event
LDLT	Living donor liver transplantation
LT	Liver transplantation
MASLD	Metabolic dysfunction-associated steatotic liver disease
mRECIST	Modified Response Evaluation Criteria in Solid Tumors
mTOR	Mechanistic target of rapamycin
OR	Odds ratio
OS	Overall survival
PD-1	Programmed cell death protein 1
PD-L1	Programmed death-ligand 1
pCCA	Perihilar cholangiocarcinoma
PRISMA-ScR	Preferred Reporting Items for Systematic Reviews and Meta-Analyses extension for Scoping Reviews
RAI	Rejection Activity Index
UNOS	United Network for Organ Sharing
